# Chronic exposure to particulate matter and risk of cardiovascular mortality: cohort study from Taiwan

**DOI:** 10.1186/s12889-015-2272-6

**Published:** 2015-09-21

**Authors:** Eva Tseng, Wen-Chao Ho, Meng-Hung Lin, Tsun-Jen Cheng, Pau-Chung Chen, Hsien-Ho Lin

**Affiliations:** Division of General Internal Medicine, Johns Hopkins University, Baltimore, MD USA; Department of Public Health, China Medical University, Taichung City, Taiwan; Institute of Occupational Medicine and Industrial Hygiene, National Taiwan University, Taipei, Taiwan; Institute of Epidemiology and Preventive Medicine, National Taiwan University, 17 Xu-Zhou Road, Rm706, Taipei, 100 Taiwan

**Keywords:** Air pollution, Particulate matter, Cardiovascular mortality, Total mortality, Cohort studies

## Abstract

**Background:**

Evidence on the association between long-term exposure to air pollution and cardiovascular mortality is limited in Asian populations.

**Methods:**

We conducted a cohort study on the association between fine particulate matter (PM_2.5_) and cardiovascular mortality using 43,227 individuals in a civil servants health service in Taiwan. Each participant was assigned an exposure level of particulate matter based on their district of residence using air pollution data collected by the Taiwan Environmental Protection Agency and with modeling using geographic information systems. The participants were followed up from 1989 to 2008 and the vital status was ascertained from death records. Cox regression models were used to adjust for confounding factors.

**Results:**

The district-level average of PM_2.5_ ranged from 22.8 to 32.9 μg/m^3^ in the study area. After a median follow-up of 18 years, 1992 deaths from all causes including 230 cardiovascular deaths occurred. After adjustment for potential confounders, PM_2.5_ levels were not significantly associated with mortality from cardiovascular disease [Hazard Ratio (HR) 0.80; 95 % Confidence Interval (CI), 0.43 to 1.50 per 10 μg/m^3^ increase in PM_2.5_] or all causes (HR 0.92; 95 % CI, 0.72 to 1.17 per 10 μg/m^3^ increase in PM_2.5_). The results were similar when the analysis was restricted to the urban areas and when the PM_2.5_ measurement was changed from the period average (2000–2008) to annual average.

**Discussion:**

Our findings are different from those in prior cohort studies conducted in Asia where ambient air pollutionwas associated with an increased risk of cardiovascular mortality. The high background level of air pollutionin our study area and the small number of event cases limited the power of this study.

**Conclusions:**

In this population-based cohort study in Taiwan, we found no evidence of increased risk for all-cause or cardiovascular mortality with long-term exposure to PM_2.5_.

**Electronic supplementary material:**

The online version of this article (doi:10.1186/s12889-015-2272-6) contains supplementary material, which is available to authorized users.

## Background

Substantial research has been performed examining the adverse health effects of air pollution, specifically fine particulate matter with a diameter of 2.5 μm or less (PM_2.5_), which is primarily produced from the combustion of fossil fuels. The size of these fine particles allows them to be deposited deep down in the alveoli of the lung, resulting in prothrombotic states, endothelial dysfunction, progression of atherosclerosis, and increased systemic oxidative stress [1, 2]. PM has been established as a trigger of cardiovascular events occurring within hours to days after exposure [3]. Moreover, studies have shown that extended exposure to fine particulate matter is an important predictor of mortality for cardiopulmonary disease [4–6]. A meta-analysis concluded that there was a 6.2 % (95 % CI, 4.1 to 8.4 %) increased risk of all-cause mortality per 10 μg/m^3^ increase in PM_2.5_ exposure and 10.6 % (95 % CI, 5.4 to 16.0 %) increased risk of cardiovascular mortality per 10 μg/m^3^ increase in PM_2.5_ [7]. The 2010 review by the American Heart Association writing group concluded that PM_2.5_ exposure is a “modifiable factor contributing to cardiovascular morbidity and mortality” [3].

The generalizability of these studies, conducted primarily in North America and Europe, to Asia is less known. Compared to Western countries, Asian countries have higher air pollution levels with different emission sources. Higher population density coupled with increasing urbanization mean that city residents in Asia have a greater exposure to air pollution than their counterparts in Western countries. Additionally, East Asian countries tend to have a lower coronary heart disease mortality but higher stroke mortality compared to Western countries [8].

Despite increasing public health interest in the effects of air pollution, attempts to provide a comprehensive global risk assessment have been limited in generalizability due to the small number of long-term cohort studies conducted in Asia. The majority of studies conducted in Asian countries have focused on short-term PM exposure [9, 10]. Few cohort studies from Asia, including those conducted in China, Japan, and Hong Kong, have studied the effects of long-term exposure to air pollution, with only one study focusing on PM_2.5_ [11–16]. Therefore, the estimates for the global burden of disease attributable to ambient air pollution relies heavily on studies from Western countries [17, 18].

Given the limited evidence on the health effects of long-term exposure to ambient air pollution in Asia, we conducted a retrospective cohort study to examine the association between PM_2.5_ and cardiovascular and all-cause mortality in Taiwan, an Asian country with high levels of ambient air pollution.

## Methods

### Study population

**Fig. 1:**
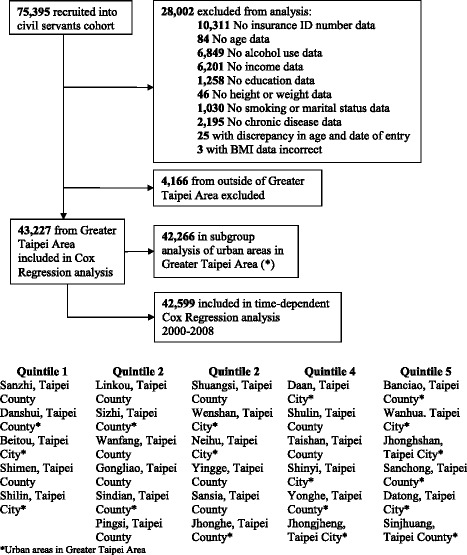
Flow diagram of exclusions and subgroup analyses of the civil servants cohort, Taiwan

The study population was derived from a cohort of 75,395 individuals who were civil service employees and teachers. These individuals underwent an annual physical examination at the Taipei Outpatient Service Center as part of the government employee insurance program from 1989 to 1992 [19, 20]. Of the 75,395 individuals enrolled in the civil servants cohort, we excluded from analysis 28,002 people with missing data on personal identification number and cardiovascular risk factors. Additionally, we excluded 4166 individuals who resided outside of Greater Taipei Area, which is defined as the area including New Taipei City and Taipei City. Within the Greater Taipei Area, 29 districts were represented out of a total of 41 districts. After these exclusions, 43,227 subjects (24,630 males and 18,597 females) were included in the final study (Fig. 1). The number of subjects, total population size, and area of each district are presented in the Appendix (see Additional file 1). The study cohort was followed from 1992 up until December 31st of 2008.

### Exposure assessment of ambient air pollution

We obtained data on air pollution levels of PM_2.5_ from the Taiwan Environmental Protection Agency’s Taiwan Air Quality Monitoring Network, which was established in 1990 and began collecting data in September 1993. The Air Quality Monitoring Network collects air pollution data from 76 stations in seven established districts. Mean pollution levels of PM_2.5_ were calculated over the period 2000–2008 and used as the primary exposure variable in the Cox regression analysis. Although PM_2.5_ measurements began in 1995, nationwide survey of PM_2.5_ was not implemented until August 2005 [21]. Therefore, the PM_2.5_ data for Greater Taipei City between 2000 and August 2005 was estimated using backward prediction based on historical trends. Specifically, we used data on other pollutants (PM with a diameter of 10 μm or less, sulfur dioxide, nitric oxide, ozone and carbon monoxide) and temperature levels to estimate station-specific levels of PM_2.5_. We then used geographic information systems to estimate the PM_2.5_ level at the district level using the station-specific data and estimates for the period 2000–2008. We did not have air pollution estimates prior to 2000 available. Therefore, we used the estimates for 2000–2008 as a proxy for ambient air pollution during the period of cohort follow up from 1992 through 2008. Each participant was assigned an average PM_2.5_ level of exposure based on their district of residence which was determined by their phone number listed on the baseline questionnaire survey. For the time-dependent Cox regression analysis, we used the annual average PM2.5 levels from 2000–2008 as the time-varying exposure.

### Measurement of covariates

Information on known cardiovascular risk factors was obtained through self-administered questionnaires at baseline, including socioeconomic and demographic data such as marital status, income, education level, alcohol consumption, smoking, and selected baseline illnesses (hypertension and diabetes). Total cholesterol and triglyceride levels were determined by fasting venous blood sampling. The abbreviated Modification of Diet in Renal Disease Study formula was used to assess glomerular filtration rate (GFR).

### Ascertainment of mortality and cause of death

The primary outcome of the study was cardiovascular and all-cause mortality. Vital status was determined by death records which are collected by the Taiwan Department of Health and have been computerized since 1971. The underlying cause of death was coded according to the International Classification of Disease 9^th^ revision (ICD-9) and 10^th^ revision (ICD-10). We used the following ICD codes to assess deaths from cardiovascular disease: ischemic heart disease (ICD-9 410–414, ICD-9 429.2, ICD-9 429.7, and ICD-10 I20-25) and cerebrovascular disease (ICD-9 430–438 and ICD-10 I60-69).

### Statistical analysis

We used Cox proportional hazards model to estimate the hazard ratio and 95 % confidence interval (CI) for all-cause and cardiovascular mortality due to ambient air pollution. The primary exposure variable was the average PM_2.5_ level over the period 2000–2008. The 29 districts in greater Taipei City that were represented in the cohort were ranked based on their average PM_2.5_ levels and then divided into quintiles with the reference group being the quintile with the lowest average. We decided to model particulate matter as a linear continuous variable and by quintiles because we wanted to assess if there may be a non-linear dose–response relationship between air pollution and cardiovascular mortality [17]. The ages of the subjects when they entered and exited the cohort were used to define the time variable for the Cox models. We adjusted for confounders by creating indicator variables for never, current or former smokers, habitual alcohol use, married or other, less then high school education, and body mass index (BMI) (with cutoffs at 20.1 and 27.5 kg/m^2^ based on a study suggesting that the lowest risk of mortality for East Asians is in that range) [22]. We did not adjust for a history of cerebrovascular disease, heart disease, diabetes and hypertension because they are potential intermediaries between air pollution and the outcome of interest. Additionally, we did not adjust for lipid levels and renal function because there is some evidence suggesting that air pollution may be predictors of lipid disorders and decreased renal function [23, 24].

We performed additional analyses using time-dependent cox regression to examine the effect of sub-chronic exposure to PM_2.5_ over the period of 2000–2008. We were concerned that averaging the PM_2.5_ levels over time may have decreased the variability of exposure. Therefore, we examined time-varying exposures of one year in duration. Participants who died prior to 2000 were excluded and the remaining cohort was followed for events between 2000 and 2008.

Additionally, we conducted a subgroup analysis of the urban areas within greater Taipei City because we were concerned if there was confounding from area-level factors such as access to medical care, assuming that people living in less urban areas would have limited access to medical care. This subgroup included a total 42,266 individuals from 18 districts that were selected by the authors and deemed as “urban” areas (Fig. 1). Finally, we also examined the effect of other air pollutants including carbon monoxide (CO), nitric oxide (NO), nitrogen dioxide (NO_2_), nitrogen oxides (NO_X_), sulfur dioxide (SO_2_), and ozone (O_3_) in several two-pollutant models. Statistical significance levels were determined by 2-sided *p* value of 0.05. All statistical analyses were carried out with SAS (Version 9.2, SAS Institute, Inc., Cary, NC).

### Standard protocol approvals, registrations, and patient consent

All participants in the study gave written informed consent. This study was approved by the Institutional Review Board of National Taiwan University.

## Results

**Fig. 2:**
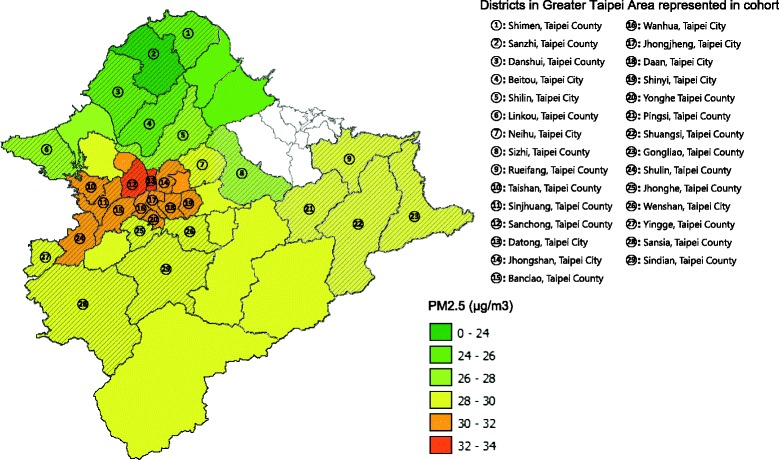
Map of districts in Greater Taipei Area with corresponding average PM_2.5_ level. The shaded area represents districts where the study participants came from

The baseline characteristics of the cohort and air pollution measures are listed in Table 1. The average PM_2.5_ levels across the five quintiles ranged from 25.8 μg/m^3^ to 32 μg/m^3^ (Fig. 2). The age and sex distributions were similar among the quintiles with women representing about 40–45 % of the cohort. Only around 2 % of participants had lower than high-school education. The majority of subjects had a monthly income between $30,000 and $100,000 New Taiwan dollars. Current smoking levels, habitual alcohol consumption, baseline total cholesterol, triglyceride and GFR measurements were also consistent across the quintiles. Baseline comorbid conditions were similar, with only about 2 % of individuals reporting a history of diabetes, 0.3 % reporting cerebrovascular disease, 9 % reporting hypertension and around 5 % reporting heart disease.

**Table 1:** Baseline characteristics of the civil servants cohort according to quintiles of average PM_2.5_ concentration during 1989–2008

Characteristic	Quintile				
	1	2	3	4	5
No. of participants	5,520	3,847	8,267	19,004	6,589
Range of average PM_2.5_ concentration, μg/m^3^	22.8–27.2	27.3–28.6	28.7–30.0	30.1–30.9	31.5–32.9
Mean PM_2.5_ in μg/m^3^ (SD)	25.8 (1.4)	28.3 (0.4)	29.6 (0.4)	30.4 (0.3)	32 (0.4)
Female sex (%)	47.3	38.7	39.2	44.5	42.6
Less than high-school education (%)	1.9	1.7	2.3	1.8	2.6
Marital status					
Married (%)	45.7	41.1	39.3	39.2	37.9
Other (%)	54.3	58.9	60.7	60.8	62.1
Income in NT (%)					
≤ $ 30,000	8.6	10.1	10.5	7.1	11.2
$30,000-60,000	45.9	52.1	51.7	43	51.7
$60,000-100,000	37.3	31.8	32.1	39.4	31.2
> $100,000	8.2	6.1	5.8	10.5	5.9
Average age at recruitment in years (SD)	40.6 (10.4)	40.6 (10.7)	40.4 (10.4)	42.5 (10.5)	39.7 (10.8)
Smokers (%)					
Never	52.2	47.4	48.4	47.7	49.7
Former	7.7	8	8.2	7.5	7
Current	40.1	44.6	43.4	44.8	43.3
Habitual alcohol consumption (%)	72.4	75.3	73.7	74.6	73
Average body-mass index^a^, %	22.7	23.1	23.9	22.8	22.8
< 20.1	19	14.8	16.7	16.9	17.9
20.1-27.5	75.1	78.6	76.6	77.7	76
> 27.5	5.9	6.7	6.7	5.4	6.1
Serum triglyceride level (mg/dL)	113	116	116	113	117
Serum total cholesterol level (mg/dL)	197	194	195	197	197
Diabetes mellitus (%)	2.2	1.8	1.7	2.1	1.7
Hypertension (%)	9	9.8	9.6	10.2	8.9
History of cerebrovascular disease (%)	0.4	0.3	0.4	0.3	0.3
History of heart disease (%)	4.4	5	5	5.2	4.5
Average GFR^b^ (mL/min/1.73 m^2^)	83	83	82.8	83.1	82.9

*GFR* glomerular filtration rate, *NT* National Taiwan dollars, *SD* standard deviation

^a^Calculated as weight in kilograms divided by height in meters squared

^b^Calculated using the modification of diet in renal disease (MDRD) equation: eGFR (mL/min/1•73 m2) = 175 × (Scr)-1•154 × (Age)-0•203 × (0•742 if female)

During the median 18-year follow-up, 280 deaths from cardiovascular disease (including 139 from ischemic heart disease and 141 from cerebrovascular disease) and 1992 deaths from all causes occurred. The crude mortality rate (all-cause and cardiovascular) did not reveal a strong dose-dependent pattern across quintiles of PM_2.5_ (Table 2). After adjusting for sex, age, education, marital status, income, smoking, alcohol consumption, and BMI, the level of PM_2.5_ was not significantly associated with all-cause (HR per 10 μg/m^3^ increase in PM_2.5_: 0.92, 95 % CI, 0.72 to 1.17) and cardiovascular mortality (HR per 10 μg/m^3^ increase in PM_2.5_: 0.80, 95 % CI, 0.43 to 1.50). There appeared to be an inverse association between PM_2.5_ and mortality in the quintile and continuous-variable analysis, but the *p* values for trend were not significant. The HRs did not change considerably in the crude versus multivariable-adjusted model (Model 1 and Model 2 in Table 2), suggesting that there was not much confounding present. Increased cardiovascular mortality was significantly associated with males (HR 2.42, 95 % CI, 1.65 to 3.56) and increased BMI > 27.5 kg/m^2^ (HR 1.90, 95 % CI, 1.08 to 3.35) and inversely associated with higher than high school education (HR 0.48, 95 % CI, 0.33 to 0.71).

**Table 2:** Multivariate-adjusted hazard ratios for all-cause, CVD, IHD and cerebrovascular mortality, estimated from cox proportional-hazards model

	No. of persons	No. of deaths	Deaths/1000 person-years	Model 1^a^	Model 2^b^
				HR (95 % CI)	HR (95 % CI)
All–causes					
Per 10 μg/m^3^ increase				0.90 (0.71–1.15)	0.92 (0.72–1.17)
Q1	5,520	246	0.77	1.00	1.00
Q2	3,847	202	0.91	1.09 (0.91–1.32)	1.10 (0.92–1.33)
Q3	8,267	367	0.77	1.01 (0.86–1.18)	0.99 (0.84–1.16)
Q4	19,004	900	0.79	0.91 (0.79–1.05)	0.95 (0.82–1.09)
Q5	6,589	277	0.74	0.99 (0.84–1.18)	0.98 (0.82–1.16)
*p*-value for linear trend					0.92
CVD					
Per 10 μg/m^3^ increase				0.78 (0.42–1.48)	0.80 (0.43–1.50)
Q1	5,520	34	0.11	1.00	1.00
Q2	3,847	31	0.14	1.23 (0.76–2.01)	1.25 (0.77–2.04)
Q3	8,267	56	0.12	1.12 (0.73–1.71)	1.07 (0.70–1.64)
Q4	19,004	121	0.11	0.87 (0.59–1.27)	0.93 (0.63–1.36)
Q5	6,589	38	0.1	0.99 (0.62–1.57)	0.94 (0.59–1.49)
*p*-value for linear trend					0.49
IHD					
Per 10 μg/m^3^ increase				0.74 (0.30–1.81)	0.76 (0.31–1.84)
Q1	5,520	15	0.047	1.00	1.00
Q2	3,847	15	0.068	1.25 (0.62–2.54)	1.26 (0.62–2.55)
Q3	8,267	33	0.069	1.42 (0.78–2.57)	1.33 (0.73–2.42)
Q4	19,004	60	0.053	0.92 (0.53–1.60)	0.99 (0.57–1.72)
Q5	6,589	15	0.04	0.84 (0.42–1.70)	0.80 (0.40–1.63)
*p*-value for linear trend					0.84
Cerebrovascular disease					
Per 10 μg/m^3^ increase				0.68 (0.34–2.04)	0.84 (0.35–2.04)
Q1	5,520	18	0.057	1.00	1.00
Q2	3,847	16	0.072	1.22 (0.62–2.39)	1.24 (0.63–2.44)
Q3	8,267	23	0.048	0.86 (0.46–1.60)	0.83 (0.45–1.54)
Q4	19,004	61	0.054	0.82 (0.48–1.38)	0.88 (0.52–1.49)
Q5	6,589	23	0.061	1.11 (0.60–2.06)	1.05 (0.56–1.94)
*p*-value for linear trend					0.76

*CVD* cardiovascular disease (combines IHD and cerebrovascular disease), *IHD* ischemic heart disease, *CI* confidence interval, *HR* hazard ratio

^a^Model 1: Adjusted for age

^b^Model 2: Adjusted for age, sex, marital status, income, smoking, alcohol, BMI, and education

In the subgroup analysis of solely the urban areas within greater Taipei City (Fig. 1), results were similar to our prior analysis in that both crude and multivariable adjusted cardiovascular and all-cause mortality were not significantly associated with air pollution (data not shown). Given our concern that using the average PM_2.5_ level as the exposure variable may not be sensitive enough to show any association, we performed a time-dependent Cox regression analysis to investigate the sub-chronic effect of annual PM_2.5_ exposure. We found that the annual level of PM_2.5_ was not associated with cardiovascular mortality (HR 0.57, 95 % CI, 0.28 to 1.18 per 10 μg/m^3^ increase in PM_2.5_). Finally, we investigated several other air pollutants including CO, NO_2_, NO, NO_X_, O_3_ and SO_2_ to see if including this additional exposure would affect cardiovascular mortality. When these pollutants were analyzed individually, we did not find significant associations between the pollutants and cardiovascular mortality (Table 3). In the two-pollutant model in which we paired PM_2.5_ with one of these additional pollutants, the association between PM_2.5_ and cardiovascular mortality remained unchanged (Table 4).

**Table 3:** Multivariable-adjusted cardiovascular mortality-rate ratios for other pollutants estimated from Cox proportional-hazards model

Pollutant	Crude	Multivariable adjusted^a^
	HR (95 % CI)	HR (95 % CI)
CO (ppm)	1.43 (0.48–4.22)	1.57 (0.55–4.50)
NO (ppb)	1.02 (0.97–1.06)	1.02 (0.98–1.07)
NO_2_ (ppb)	0.99 (0.95–.103)	0.99 (0.95–1.04)
NO_X_ (ppb)	1.00 (0.98–1.02)	1.00 (0.98–1.03)
O_3_ (ppb)	1.01 (0.97–1.06)	1.00 (0.96–1.05)
SO_2_ (ppb)	0.99 (0.72–1.37)	0.91 (0.67–1.25)

*CO* carbon monoxide, *NO* nitric oxide, *NO*_*2*_ nitrogen dioxide, *NO*_*X*_ nitrogen oxides, *SO*_*2*_ sulfur dioxide, *O*_*3*_ ozone, *CI* confidence interval, *HR* hazard ratio

^a^Adjusted for age, sex, marital status, income, smoking, alcohol, BMI, and education

**Table 4:** Multivariable-adjusted cardiovascular mortality-rate ratios for PM_2.5_ after adjusting for other pollutants

Quintile	PM_2.5_	PM_2.5_ + CO	PM_2.5_ + NO_2_	PM_2.5_ + NO	PM_2.5_ + NO_X_	PM_2.5_ + O_3_	PM_2.5_ + SO_2_
	HR^a^ (95 % CI)	HR^a^ (95 % CI)	HR^a^ (95 % CI)	HR^a^ (95 % CI)	HR^a^ (95 % CI)	HR^a^ (95 % CI)	HR^a^ (95 % CI)
1	1.00 (reference)	1.00 (reference)	1.00 (reference)	1.00 (reference)	1.00 (reference)	1.00 (reference)	1.00 (reference)
2	1.25 (0.77–2.04)	1.20 (0.74–1.96)	1.05 (0.59–1.89)	1.37 (0.83–2.24)	1.17 (0.72–1.91)	0.99 (0.48–2.04)	1.32 (0.76–2.29)
3	1.07 (0.70–1.64)	0.88 (0.56–1.38)	0.82 (0.43–1.59)	1.02 (0.66–1.56)	0.86 (0.54–1.38)	0.80 (0.36–1.76)	1.13 (0.70–1.89)
4	0.93 (0.63–1.36)	0.68 (0.43–1.07)	0.65 (0.29–1.44)	0.79 (0.53–1.17)	0.65 (0.39–1.08)	0.66 (0.26–1.60)	0.99 (0.61–1.61)
5	0.94 (0.59–1.49)	0.60 (0.33–1.09)	0.63 (0.25–1.57)	0.71 (0.42–1.20)	0.60 (0.31–1.14)	0.66 (0.26–1.67)	1.04 (0.52–2.07)

*CO* carbon monoxide, *NO* nitric oxide, *NO*_*2*_ nitrogen dioxide, *NO*_*X*_ nitrogen oxides, *SO*_*2*_ sulfur dioxide, *O*_*3*_ ozone, *CI* confidence interval, *HR* hazard ratio

^a^Adjusted for age, sex, marital status, income, smoking, alcohol, BMI, and education

## Discussion

Although there have been many epidemiologic studies in Asia assessing the health effects of short-term air pollution exposure, our study represents one of only a few cohort studies examining the association between long-term exposure to PM_2.5_ and cardiovascular mortality in Asia. After extensive adjustment for confounding factors, we did not observe an association between long-term exposure to particulate air pollution and cardiovascular and all-cause mortality in our study population.

Our findings are different from those in prior cohort studies conducted in Asia. Two cohort studies, one conducted in Shenyang and the other in 4 cities in Northern China, found that both all-cause and cardiovascular mortality were associated with increasing PM_10_ levels [11, 14]. Similar results were found in another prospective cohort study conducted in urban areas across China [12]. Another Chinese cohort study demonstrated a significant association between cardiovascular mortality and exposure to total suspended particles, SO_2_ and NO [16]. Finally, a recent cohort study conducted in Hong Kong assessed the long-term effects of PM_2.5_ exposure, which was estimated using U.S. NASA satellite data, and found a significant association with both all-cause and cardiovascular mortality [13].

Our results may vary from other studies because of the limited range in air pollution exposure within our cohort, which was conducted in the Greater Taipei Area. Several studies conducted within cities, such as the Vancouver and Ontario cohorts, have found similar null results suggesting that within city studies may not be comparable with between-city studies given limited spatial variability in PM_2.5_ [25–28]. In the Netherlands cohort study, PM_2.5_ exposure was limited in range similar to our study, and the authors did not find an association between PM_2.5_ and all-cause or cardiovascular mortality [28]. In the ACS intra-urban analysis of the New York City region, PM_2.5_ was also not associated with all-cause mortality [26]. Therefore, if we had compared our cohort from the Greater Taipei Area with other cities in Taiwan, our results may be different.

Another possibility for our negative result is confounding by neighborhood socioeconomic status, since cardiovascular mortality tends to be higher in rural areas (with lower air pollution) than in urban areas (with higher air pollution). However, when we restricted our analysis to urban districts, the association between PM_2.5_ and all-cause and CVD mortality remained unchanged. In addition, the difference between our study population and those in previously published studies should be noted. For example, the Harvard Six Cities Study population had a higher percentage of smokers (combining former and current smokers) and lower education levels, and many of the participants were exposed to dust or fumes at their job. In contrast, our study population only includes civil servants who have a high education level, steady income and likely no occupational exposure to dust or fumes. Therefore, their stable socioeconomic status may confer them less vulnerability to air pollution as suggested by prior published studies which argue that people with a high socioeconomic status and healthier lifestyles are less susceptible to the cardiovascular effects of air pollution [29].

We found a non-significant inverse trend between particulate matter and cardiovascular mortality. Our results are similar to the NIPPON DATA80 Japanese cohort study which found an inverse association between cardiovascular mortality and PM although their findings were statistically significant [15]. In this cohort study of 7250 individuals from 300 randomly selected districts in Japan, the authors found that adjusted HRs for cardiovascular mortality tended to decrease per 10 μg/m^3^ increase in PM (HR 0.90, 95 % CI, 0.81 to 1.00). This inverse trend was also demonstrated in the Netherlands cohort in their case-cohort analysis after extensive adjustment for confounders, although the trend was not statistically significant [28].

The strengths of our study include having 20 years of prospective follow-up of a large cohort. We were able to adjust for confounders including cardiovascular risk factors like smoking and individual socioeconomic variables like income and education. We did several subsequent analyses including time-dependent cox regression analysis to see if sub-chronic exposure to PM_2.5_ would be a more sensitive measure of the exposure variability. We also examined the effect of other pollutants on the association between PM_2.5_ and CVD mortality. Evidence shows that these pollutants may have independent adverse effects on cardiovascular risk [8].

There are several limitations to our study. First, a major limitation was the lack of power due to the small number of event cases. This may in part be due to our use of ICD codes to determine the cause of death which can potentially lead to the underreporting of deaths due to cardiovascular disease and underestimate the effects. Second, studies conducted in Asia that found a positive association between cardiovascular mortality and PM defined cardiovascular deaths much more broadly than we did by including a wider range of ICD codes which could have led to a significant association [9, 12, 13]. Third, since our study is confined to the Taipei metropolitan basin, there was limited variability in PM_2.5_ exposure which may have restricted our ability to detect a significant association. Fourth, we used only the participant’s phone number to assign them to a residential district and did not account for the possibility that participants moved during the study. Additionally, the air pollution data we used was partially obtained by backward prediction. Thus, both limitations could have led to exposure misclassification. Finally, we adjusted for personal characteristics only at baseline.

## Conclusions

In this large population of civil servants from an Asian city, we did not find an increased risk of cardiovascular mortality with long-term exposure to fine particulate matter. Our findings were limited due to the small number of event cases and spatial variability in PM_2.5_. Nevertheless, given the paucity of data in Asia, we believe our study adds further knowledge to the current body of literature and highlights the need for more cohort studies to accurately estimate the effects of long-term air pollution on mortality in Asia.

## Additional file

Additional file 1:
**Population size, area and cohort representation by districts in the Greater Taipei Area, Taiwan.** (XLSX 10 kb)

## Abbreviations

BMI: Body mass index
CO: Carbon monoxide
CVD: Cardiovascular and cerebrovascular disease
GFR: Glomerular filtration rate
ICD: International classification of disease
IHD: Ischemic heart disease
NO_2_: Nitrogen dioxide
NO: Nitric oxide
NO_X_: Nitrogen oxides
NT: National Taiwan dollars
O_3_: Ozone
PM_2.5_: Particulate matter with a diameter of 2.5 μm or less
PM_10_: Particulate matter with a diameter of 10 μm or less
SO_2_: Sulfur dioxide
